# First Isolation and Genomic Characterization of BVDV-1c in Przewalski’s Gazelle (*Procapra przewalskii*) from the Qinghai–Tibet Plateau, China

**DOI:** 10.3390/ani16172781

**Published:** 2026-09-04

**Authors:** Yue Zhang, Yang Zhang, Guanghua Wang, Xiuping Li, Yong Hu, Wen Guo, Yutong Liu, Linyuan Gong, Xingjun Wang, Mingxue Wang, Lili Huo, Yingna Jian, Liqing Ma

**Affiliations:** 1 Qinghai Provincial Key Laboratory of Pathogen Diagnosis for Animal Diseases and Green Technical Research for Prevention and Control, Academy of Animal Sciences and Veterinary, Qinghai University, Xining 810016, China; zhang_yue_1995@163.com (Y.Z.);; 2 Qinghai Lake Biodiversity Conservation Research Center, Gonghe 813099, China

**Keywords:** *Procapra przewalskii*, bovine viral diarrhea virus (BVDV), BVDV-1c, phylogenetic analysis, cross-species transmission, disease surveillance

## Abstract

Przewalski’s gazelle (*Procapra przewalskii*) is an endangered ungulate endemic to the Qinghai–Tibet Plateau of China. Increasing habitat alteration and close contact with domestic livestock have raised concerns about cross-species pathogen transmission, yet infectious disease studies in this species remain limited. To determine the etiology of illness in two deceased gazelles from a conservation facility in Qinghai Province, we screened samples for a panel of pathogens, including *Mycoplasma ovipneumoniae*, *Clostridium perfringens* toxin genes, *Mannheimia haemolytica*, *Klebsiella pneumoniae*, *Mycoplasma capricolum* subsp. *capripneumoniae*, *Pasteurella multocida*, *Peste des petits ruminants virus* (PPRV), *Bovine viral diarrhea virus* (BVDV), and *Infectious bovine rhinotracheitis virus* (IBRV), using PCR and RT-PCR. BVDV-specific nucleic acids were detected in tissue samples from both individuals, whereas all other targeted pathogens tested negative. The virus was successfully isolated in Madin–Darby Bovine Kidney (MDBK) cells and confirmed by RT-PCR, followed by whole-genome sequencing of the isolate, which was designated QH PSYL 2026. Phylogenetic analysis based on the full-length genome and 5′UTR sequences assigned the isolate to the BVDV-1c subgenotype. Notably, its 5′UTR sequence shared 100% identity with those of local cattle-derived BVDV strains, providing molecular evidence suggestive of an epidemiological linkage between wildlife and livestock. Integrating clinical signs, gross pathology, and laboratory results, the cases were consistent with BVDV infection as the primary presumptive etiology. To our knowledge, this is the first report of BVDV infection, virus isolation, and genomic characterization in Przewalski’s gazelle. The detection of a BVDV-1c strain in this endangered species highlights the potential threat that livestock-associated pathogens pose to wildlife on the Qinghai–Tibet Plateau. These findings furnish crucial baseline data for disease surveillance, molecular epidemiology, and conservation management of Przewalski’s gazelle and provide valuable scientific evidence for wildlife disease prevention and control in plateau ecosystems.

## Simple Summary

Przewalski’s gazelle is a rare animal found only on the Qinghai–Tibet Plateau, with a very small population. Its habitat is shrinking, and it increasingly comes into contact with farm animals, raising concerns that diseases may pass from livestock to wildlife. However, little is known about infections in this species. We examined two gazelles that died at a conservation center and found both were infected with bovine viral diarrhea virus, a common cattle virus. We isolated the virus and determined its genetic sequence, showing that it belongs to type 1c. The viral sequence from the gazelles was almost identical to those from local yaks and cattle, suggesting the virus may have originated from livestock. This is the first time this virus has been found in Przewalski’s gazelle. Our results emphasize the need to monitor diseases at the wildlife–livestock interface for better conservation.

## 1. Introduction

Przewalski’s gazelle (*Procapra przewalskii*) is an endangered ungulate species endemic to China and is listed as a national first-class protected wild animal. Historically widespread around Qinghai Lake and in adjacent areas of Gansu and Inner Mongolia, the species has experienced a dramatic population decline due to overhunting, habitat fragmentation, anthropogenic disturbance, and infectious diseases [1,2,3]. Although intensive conservation efforts—including the establishment of nature reserves, captive breeding, translocation, and ecological restoration—have facilitated partial population recovery, the current population remains small, genetically depauperate, and ecologically vulnerable. Consequently, infectious diseases continue to pose a persistent threat to the long-term viability of this species [4].

The rapid expansion of livestock production on the Qinghai–Tibet Plateau, coupled with the increasing spatial overlap between pastoral areas and wildlife habitats, has intensified contact between wild ungulates and domestic ruminants, thereby elevating the risk of cross-species pathogen transmission [5]. Accumulating evidence indicates that multiple livestock-associated pathogens are capable of infecting wild ungulates and may establish novel sylvatic maintenance cycles, creating challenges for both wildlife conservation and livestock disease control [6,7]. Thus, systematic surveillance of infectious diseases in endangered wildlife is essential for elucidating transmission dynamics and informing evidence-based management strategies [8].

Bovine viral diarrhea virus (BVDV), a member of the genus *Pestivirus* within the family Flaviviridae, is a globally significant pathogen of domestic ruminants. BVDV infection manifests clinically as fever, diarrhea, mucosal erosion, reproductive failure, and immunosuppression, frequently predisposing animals to secondary infections. Additionally, the virus can establish persistent infection (PI) in immunotolerant animals, which serve as efficient reservoirs for horizontal transmission, resulting in substantial economic losses worldwide [9,10,11]. Beyond cattle and sheep, BVDV exhibits a remarkably broad host range and has been documented in diverse wild ungulates, including cervids, wild sheep, and various antelope species [10,11]. Serological and molecular evidence has confirmed BVDV exposure or infection in bison, elk, wild sheep, and several captive antelope populations, suggesting that wildlife may participate in the maintenance and dissemination of the virus [12,13]. However, no information is currently available regarding the susceptibility of Przewalski’s gazelle to BVDV or the clinical outcomes of such infections.

In addition to BVDV, other pathogens—including infectious bovine rhinotracheitis virus (IBRV), peste des petits ruminants virus (PPRV), and several bacterial respiratory pathogens—are known to cause severe respiratory and gastrointestinal diseases in ruminants and may contribute to morbidity and mortality in wildlife populations [14,15,16]. Therefore, a comprehensive multi-pathogen screening approach is indispensable for accurate etiological diagnosis during disease outbreaks in wild ruminants.

In May 2026, two adult *Przewalski’s gazelles* (one male and one female) were found dead at the Qinghai Przewalski’s gazelle Conservation Research Center. These free-ranging animals were maintained under natural grazing conditions, with domestic sheep and yaks grazing in adjacent overlapping pastures with frequent interspecies contact. The remaining gazelles in the same herd showed no clinical abnormalities. Despite the recognized susceptibility of various wild ungulates to common ruminant pathogens, the pathogen profiles and disease risks specific to Przewalski’s gazelle remain largely uncharacterized, and long-term epidemiological monitoring data between sympatric livestock and gazelles are absent, leading to unclear cross-species transmission associations. Therefore, we performed postmortem examinations and molecular screening for a panel of nine viral and bacterial pathogens—including *Mycoplasma ovipneumoniae*, *Clostridium perfringens* toxin genes, *Mannheimia haemolytica*, *Klebsiella pneumoniae*, *Mycoplasma capricolum* subsp. *capripneumoniae*, *Pasteurella multocida*, PPRV, BVDV, and IBRV—to identify potential infectious causes of mortality and to generate essential baseline data for the conservation management of this critically endangered species.

## 2. Materials and Methods

### 2.1. Sample Processing

Necropsies were performed on the two deceased Przewalski’s gazelles in the laboratory. Gross lesions in the liver, spleen, lung, kidney, and intestine were recorded and photographed. Tissue samples from each organ were collected aseptically using sterile instruments; a small portion of each was transferred into a sterile 1.5 mL tube for nucleic acid extraction, and the remaining tissues were stored at −80 °C for potential further analyses.

### 2.2. Extraction of DNA and RNA

Total viral DNA and RNA were extracted separately using the Virus DNA Kit and Virus RNA Kit (TIANGEN Biotech, Beijing, China), respectively, according to the manufacturers’ instructions. Briefly, 200 μL of each sample was mixed with lysis buffer and incubated thoroughly. The lysate was then transferred to an adsorption column, washed twice, and finally eluted with 50 μL of nuclease-free water to obtain the purified nucleic acids. The extracted DNA and RNA were stored at −20 °C until use.

### 2.3. Reverse Transcription of RNA into cDNA

Reverse transcription was carried out using the PrimeScript™ RT Master Mix kit (Takara Bio Inc., Shiga, Japan). The 20 μL reaction mixture consisted of 8 μL of extracted nucleic acid, 4 μL of 5× RT buffer, 1 μL of reverse transcriptase mix, 1 μL of random primers (50 μmol/L), and nuclease-free water to volume. The reverse transcription conditions were 37 °C for 15 min followed by 85 °C for 5 s. The synthesized cDNA was subsequently used for PCR amplification.

### 2.4. PCR and RT-PCR Detection of Pathogens

PCR amplification was performed in a 25 μL reaction system. For RNA viruses (BVDV), 5 μL of cDNA template was used, whereas for DNA viruses and bacterial pathogens, 5 μL of extracted nucleic acid was used as template. Each reaction mixture contained 12.5 μL of 2 × Taq PCR Master Mix (TransGen Biotech, Beijing, China), 0.5 μL each of forward and reverse primer (10 μmol/L), and nuclease-free water to a final volume of 25 μL.

The thermal cycling conditions were as follows: initial denaturation at 94 °C for 3 min, followed by 35 cycles of denaturation at 94 °C for 30 s, annealing at the specific temperature indicated in Table 1 for each primer pair for 30 s, and extension at 72 °C for 30 s, with a final extension at 72 °C for 7 min. Positive controls (known positive samples for target pathogen) and negative controls (nuclease-free water) were included in each PCR assay. All primers used in this study are listed in Table 1.

### 2.5. Agarose Gel Electrophoresis

Five microliters of PCR products were subjected to electrophoresis on 1.5% agarose gels at 120 V for approximately 35 min. After electrophoresis, gels were visualized and photographed using a gel imaging system, and positive results were determined according to the expected target band size.

### 2.6. Indirect Immunofluorescence Assay (IFA)

MDBK cells were seeded in 6-well plates to 70–80% confluence, washed with PBS (Solarbio, Beijing, China), and inoculated with 1 mL of the third-blind-passage supernatant plus 1 mL of DMEM (Solarbio, Beijing, China) containing 10% FBS (Gibco, Grand Island, NY, USA). After 2 h of adsorption, the medium was replaced with 5% FBS DMEM and incubated for 5 days. The monolayers were then washed with PBST, fixed with 4% paraformaldehyde for 20 min, blocked with 5% BSA for 1 h, and incubated with primary antibody (BVDV-positive serum derived from yak, which was isolated and confirmed as positive in our laboratory) overnight at 4 °C. Following washes, the cells were incubated with FITC-conjugated goat anti-mouse IgG secondary antibody for 1 h at 37 °C in the dark, washed again, and observed under a fluorescence microscope.

### 2.7. Detection of PPRV by Quantitative Real-Time PCR

For PPRV detection, a one-step quantitative real-time RT-PCR was performed using the Leagene PPRV Real-Time RT-PCR Kit (Qingdao Lijian Bio-Tech Co., Ltd., Qingdao, China) according to the manufacturer’s instructions. Each 25 μL reaction contained 12.5 μL of 2× master mix, 1 μL of enzyme mix, specific primers and a TaqMan probe targeting the N gene, and 5 μL of RNA template. The cycling protocol was reverse transcription at 50 °C for 15 min, initial denaturation at 95 °C for 3 min, followed by 40 cycles of 95 °C for 15 s and 60 °C for 30 s, with fluorescence signal collected at 60 °C. Positive and negative controls were included in each run.

### 2.8. Virus Isolation

Virus isolation was performed using Madin–Darby bovine kidney (MDBK) cells. The cells were cultured in Dulbecco’s modified Eagle’s medium (DMEM) supplemented with 10% fetal bovine serum (FBS;), 100 U/mL penicillin, and 100 µg/mL streptomycin (Solarbio, Beijing, China), and maintained at 37 °C in a 5% CO_2_ incubator. Both the MDBK cells and the FBS used in this study were confirmed to be free of BVDV by RT-PCR and virus isolation prior to use.

Small amounts of RT-PCR BVDV-positive tissues (liver, spleen, lung, kidney, and intestine) from each gazelle were separately pooled per individual animal. Tissue samples (liver, spleen, lung, kidney, and intestine) from the two deceased gazelles were homogenized in PBS (10%, *w*/*v*), centrifuged at 12,000× *g* for 10 min at 4 °C, and the supernatants were filtered through a 0.22 µm filter (Millipore, Burlington, MA, USA). Confluent MDBK cells in 6-well plates were inoculated with 500 µL of each tissue filtrate and incubated at 37 °C for 1 h for virus adsorption. Following adsorption, the inoculum was removed, and 2 mL of DMEM containing 2% FBS was added. The cultures were incubated at 37 °C and observed daily for cytopathic effect (CPE) for up to 5 days. Blind passages were performed up to three times; for each passage, cell lysates and supernatants were harvested, clarified by centrifugation, and 500 µL of the supernatant was used to inoculate fresh MDBK monolayers, with characteristic features including cell rounding, cytoplasmic granulation, nuclear pyknosis, and detachment from the monolayer. Notably, the two viral isolates shared identical 5′UTR sequences. Therefore, one isolate was randomly selected and designated QH-PSYL-2026 for subsequent viral identification and whole-genome sequencing analysis.

### 2.9. Library Construction and Whole-Genome Sequencing of Viral Genome

Next-generation sequencing (NGS) libraries were prepared according to the manufacturer’s protocol. Briefly, 200 μg of genomic DNA was randomly sheared using a Covaris system to generate fragments with an average size of 300–350 bp. The fragmented DNA was then subjected to end-repair, 5′ phosphorylation, and 3′-adenylation using the End Prep Enzyme Mix, followed by adaptor ligation to both ends. Adaptor-ligated fragments were size-selected using DNA Cleanup beads. Each library was subsequently amplified by PCR for 8 cycles with P5 and P7 primers; both primers contained sequences complementary to the flow cell for bridge PCR, and the P7 primer included a six-base index to enable multiplexing. The resulting PCR products were purified and assessed for quality using an Agilent 2100 Bioanalyzer (Agilent Technologies, Santa Clara, CA, USA). Qualified libraries were sequenced on the Illumina HiSeq X Ten, NovaSeq, or MGI2000 system in paired-end 150 bp (PE150) mode.

After quality control, the sequencing reads were assembled using Velvet (version 1.2.10), Novoplasty (version 2.7.2), A5-MiSeq (version 20160825), or SPAdes (version 3.15.4), and the optimal assembly results were selected. Gaps in the draft assemblies were filled with SSPACE (version 3.0) and GapFiller (version 1–10). Finally, Pilon (version 1.22) was employed for assembly error correction [27,28,29].

### 2.10. Phylogenetic Analysis

Phylogenetic analyses were performed separately based on the full-length genome and 5′UTR sequences using MEGA 11 (v11.0.13). Multiple sequence alignment was conducted with the built-in MUSCLE algorithm. The best-fit nucleotide substitution model was selected via the Find Best DNA Models (ML) tool according to the Bayesian Information Criterion (BIC). The GTR+G+I model was applied for full-genome sequences, and the TN93 model was used for the 5′UTR region. Maximum likelihood (ML) phylogenetic trees were constructed with 1000 bootstrap replicates, and bootstrap values ≥ 70% were considered reliable. Gaps and missing data were processed using the partial deletion method with a 95% site coverage cutoff. Representative BVDV-1 and BVDV-2 reference sequences with known subgenotypes and geographic origins were included for tree construction.

## 3. Results

### 3.1. Necropsy Findings

**Figure 1:**
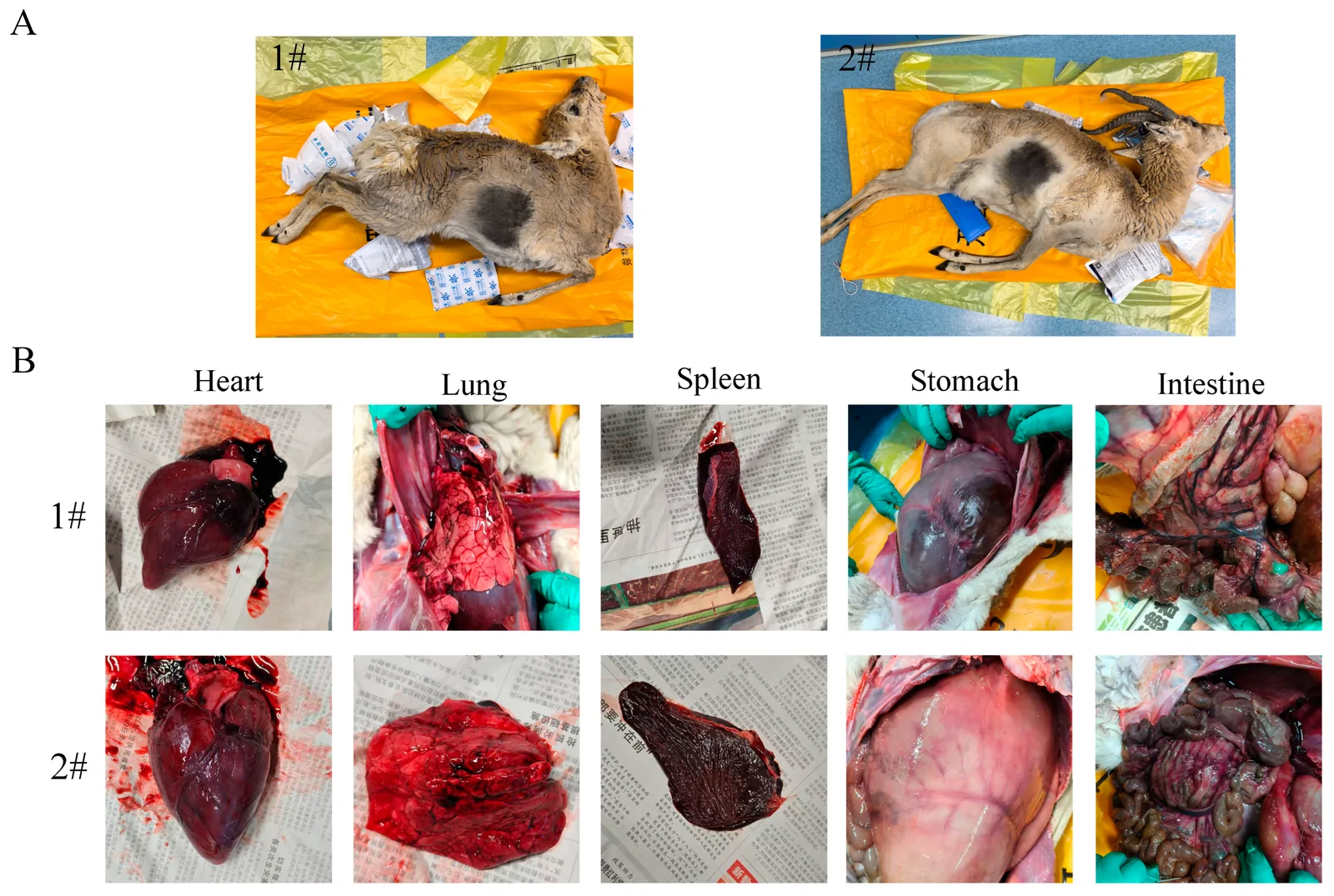
Gross appearance and necropsy findings of two deceased *Procapra przewalskii*. (**A**) External appearance of the two deceased *Procapra przewalskii* prior to necropsy. (**B**) Representative gross pathological lesions observed during necropsy, including changes in the heart, lungs, spleen, stomach, and intestines of cases No. 1# and No. 2#.

Systematic postmortem examinations were performed on the two deceased *Procapra przewalskii*, and prominent gross pathological alterations were observed in both individuals (Figure 1). In case No. 1, the principal lesions comprised severe pulmonary congestion, accompanied by hemorrhage in the auricle of the heart, marked congestion of the gastric wall, and mesenteric hemorrhage. In case No. 2, pulmonary congestion was similarly evident, along with gastric hemorrhage and multifocal gastric ulceration. No significant gross abnormalities were detected in the liver, spleen, kidneys, or other major organs.

Collectively, the predominant pathological changes involved the respiratory and gastrointestinal tracts, manifesting as pulmonary congestion and hemorrhagic lesions of the stomach and mesentery. These findings were highly suggestive of an infectious etiology, prompting further comprehensive etiological investigation.

### 3.2. Detection of Mycoplasma ovipneumoniae and Clostridium perfringens Toxin Genes

**Figure 2:**
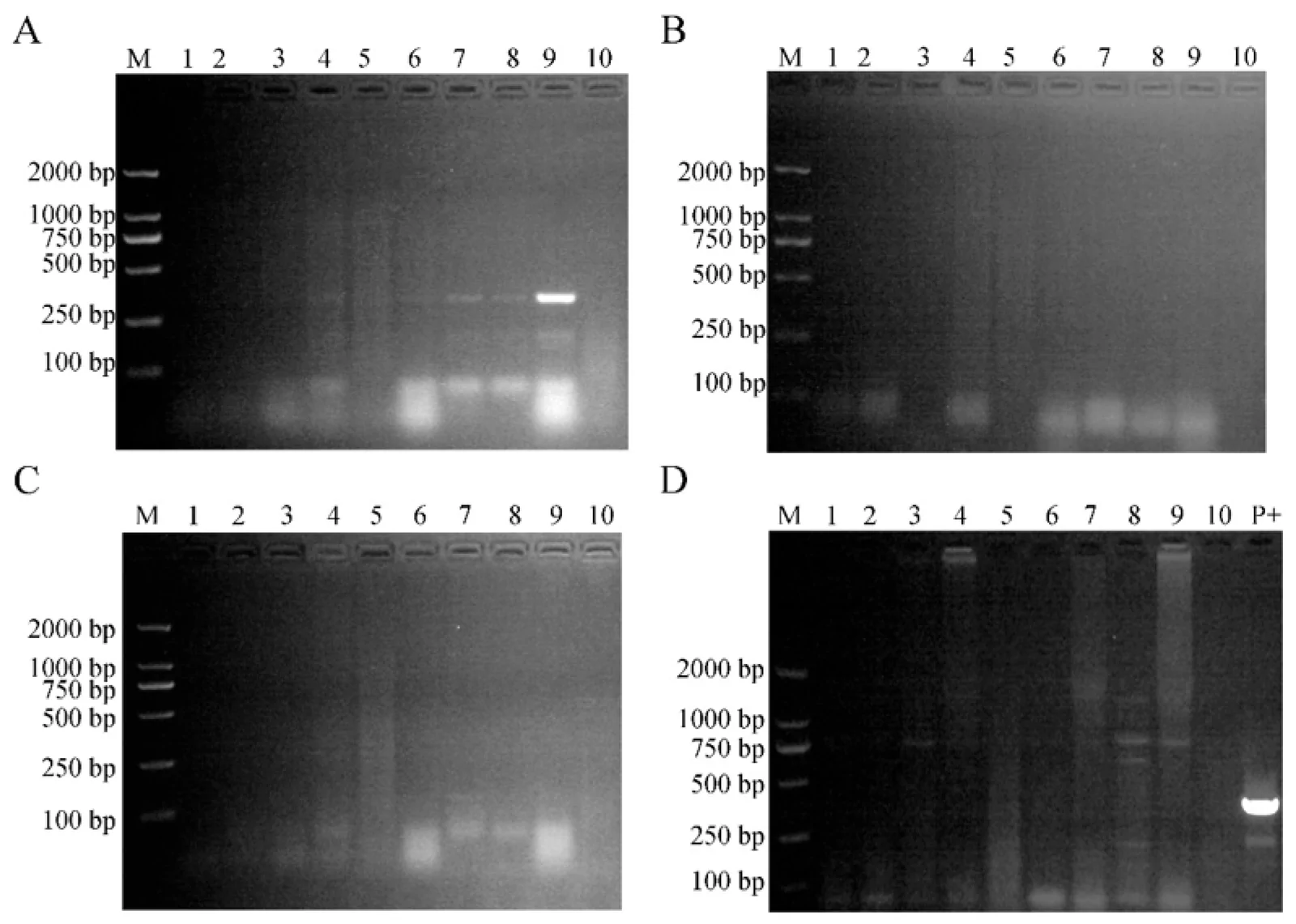
PCR detection of *Clostridium perfringens* toxin genes and *Mycoplasma ovipneumoniae* in tissue samples from two deceased Przewalski’s gazelles. (**A**) Detection of the alpha toxin gene (*cpa*) of *Clostridium perfringens*; (**B**) Detection of the beta toxin gene (*cpb*); (**C**) Detection of the epsilon toxin gene (*etx*); and (**D**) detection of *Mycoplasma ovipneumoniae*. M, DNA marker; P, positive control. Lanes 1–5 represent liver, spleen, lung, kidney, and intestine samples from *Procapra przewalskii* No. 1#, respectively; lanes 6–10 represent liver, spleen, lung, kidney, and intestine samples from *Procapra przewalskii* No. 2#, respectively.

To further investigate the cause of death in Przewalski’s gazelle, PCR assays were performed to detect *Mycoplasma ovipneumoniae* and *Clostridium perfringens* toxin genes in tissue samples. The results showed that the alpha toxin gene (*cpa*) of *Clostridium perfringens* was detected in several tissue samples, whereas the beta toxin gene (*cpb*), epsilon toxin gene (*etx*), and *Mycoplasma ovipneumoniae* were not detected. Notably, the *cpa* gene was also detected in blood samples collected from the concurrently grazing sheep flocks, yet no mortality or overt clinical signs were observed in these sheep. Therefore, additional pathogen screening was performed to identify the causative agent responsible for the fatalities. These findings indicate the presence of *Clostridium perfringens* carrying the alpha toxin gene in the examined tissues, while no evidence of infection with *Mycoplasma ovipneumoniae* or toxigenic strains harboring the *cpb* or *etx* genes was found (Figure 2). Given that the alpha toxin gene was also detected in asymptomatic sheep co-grazing with the *Procapra przewalskii*, it is unlikely that this finding accounts for the deaths observed in the *Procapra przewalskii*.

### 3.3. Detection of Mannheimia haemolytica, Klebsiella pneumoniae, Mycoplasma capricolum subsp. capripneumoniae, and Pasteurella multocida

**Figure 3:**
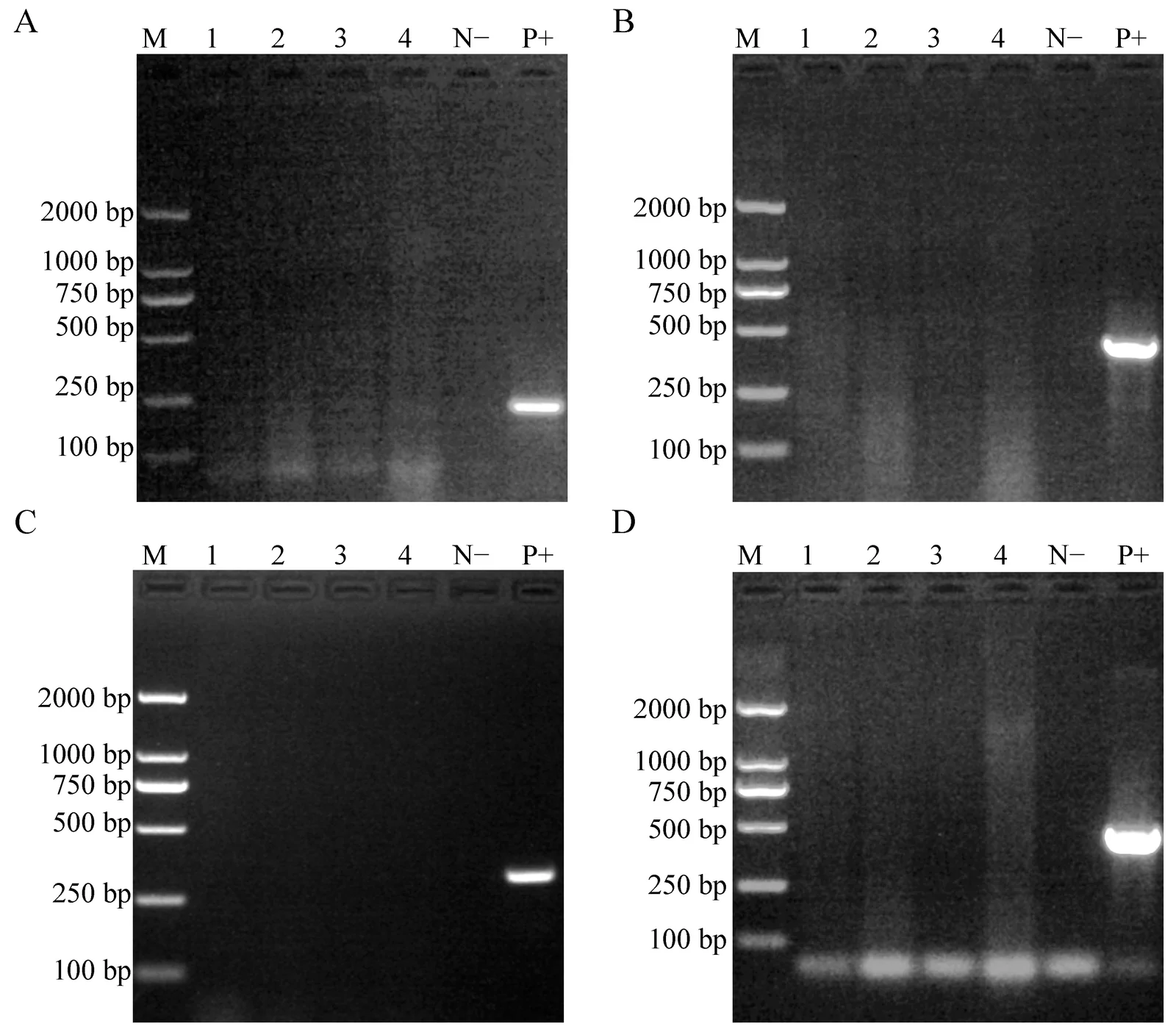
PCR detection of bacterial respiratory pathogens in tissue samples from two deceased *Procapra przewalskii*. (**A**) Detection of *Mannheimia haemolytica*; (**B**) detection of *Klebsiella pneumoniae*; (**C**) detection of *Mycoplasma capricolum* subsp. *capripneumoniae*; and (**D**) detection of *Pasteurella multocida*. M, DNA marker; N, negative control; P, positive control. Lanes 1 and 2 represent lung and spleen samples from gazelle No. 1#, respectively; lanes 3 and 4 represent lung and spleen samples from gazelle No. 2#, respectively.

Given the prominent pulmonary congestion and hemorrhagic lesions observed during necropsy, which are characteristic of severe respiratory involvement, lung and spleen tissues were selected for bacterial pathogen screening. PCR assays employing species-specific primers were performed to detect *Mannheimia haemolytica*, *Klebsiella pneumoniae*, *Mycoplasma capricolum* subsp. *capripneumoniae*, and *Pasteurella multocida*—four major bacterial pathogens that are commonly implicated in respiratory and systemic infections of domestic and wild ruminants worldwide. No specific amplification products corresponding to any of these pathogens were obtained from any of the samples (Figure 3). The absence of these bacterial agents was particularly noteworthy given the severity of the pulmonary lesions, suggesting that the observed respiratory pathology was not attributable to these common bacterial etiologies. Collectively, these findings indicated that the major bacterial pathogens commonly implicated in respiratory diseases of domestic ruminants were not involved in the mortality of the two *Procapra przewalskii*, thereby directing subsequent investigations toward potential viral etiologies.

### 3.4. Detection of PPRV, BVDV, and IBRV

**Figure 4:**
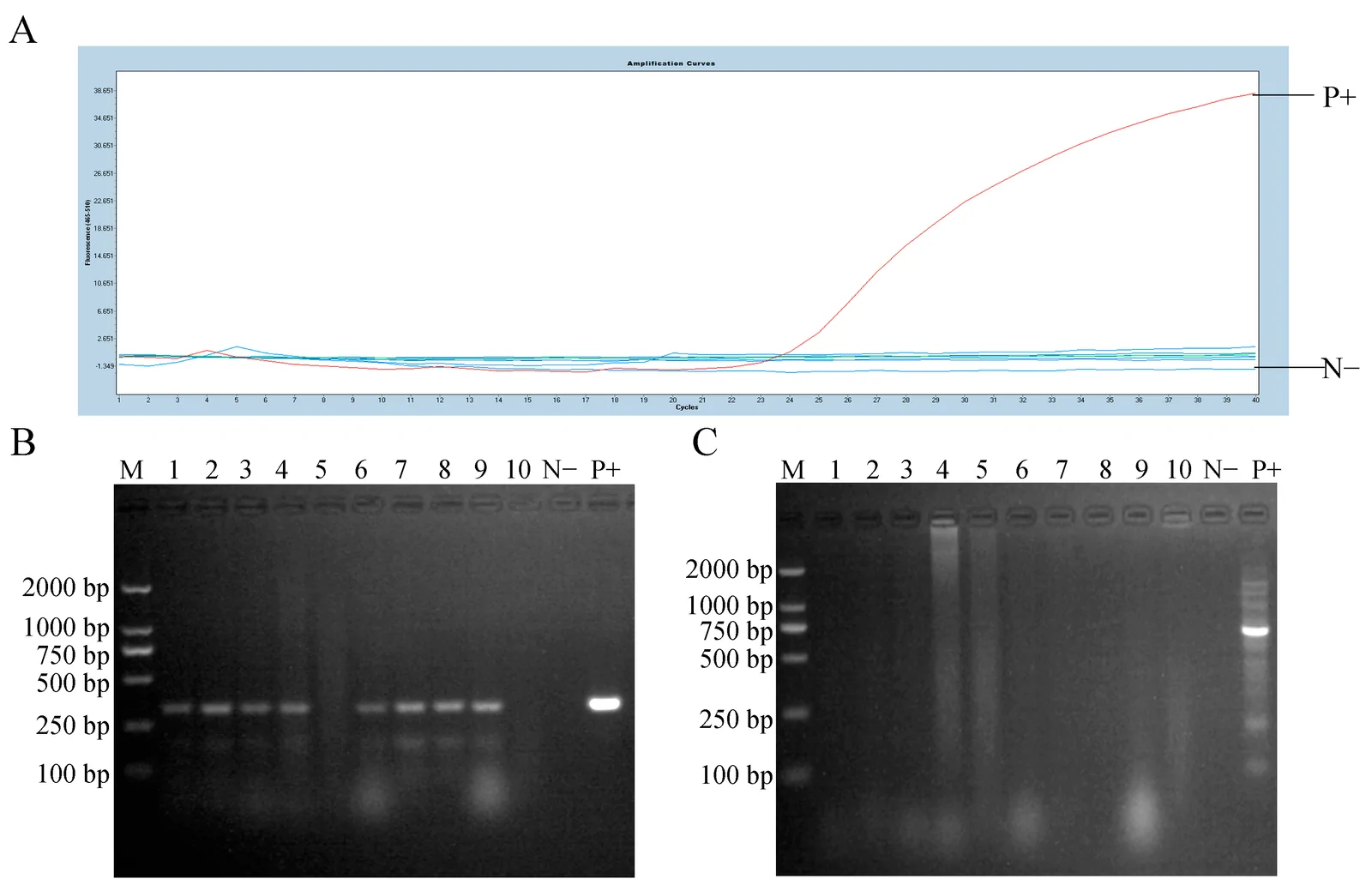
Detection of viral pathogens in tissue samples from two deceased *Procapra przewalskii*. (**A**) Real-time PCR detection of PPRV; (**B**) PCR detection of BVDV; (**C**) PCR detection of IBRV. M, DNA marker; N−, negative control; P+, positive control. Lanes 1–5 represent liver, spleen, lung, kidney, and intestine samples from gazelle No. 1, respectively; lanes 6–10 represent liver, spleen, lung, kidney, and intestine samples from gazelle No. 2, respectively.

Given the negative results for the major bacterial pathogens commonly associated with respiratory disease, further molecular screening was directed toward potential viral etiologies. Considering that PPRV, BVDV, and IBRV are among the most important viral pathogens affecting domestic and wild ruminants globally, and have been documented to cause epizootic outbreaks with high morbidity and mortality in susceptible populations, pooled tissue samples from the two deceased gazelles were subjected to viral pathogen detection using PCR and RT-PCR assays. Quantitative real-time PCR was performed to detect PPRV, and no PPRV nucleic acid was detected in any of the samples, effectively ruling out this highly contagious pathogen. Subsequently, PCR assays were conducted for BVDV and IBRV. The results revealed that BVDV-specific amplification products were successfully obtained from the tissue samples, with clear target bands of the expected molecular size, whereas no amplicons were detected for IBRV (Figure 4). The positive detection of BVDV nucleic acid, combined with the absence of other screened viral and bacterial pathogens, strongly implicated BVDV as the leading causative candidate. Moreover, the detection of BVDV-specific RNA in tissue samples, in conjunction with the prominent respiratory and gastrointestinal lesions observed at necropsy, provided compelling evidence that BVDV infection was etiologically linked to the mortality events. Collectively, based on the necropsy findings and molecular biological results, the deaths of the two *Procapra przewalskii* were preliminarily diagnosed as BVDV-associated mortality, with further virological characterization—including virus isolation and genomic sequencing—undertaken to confirm and extend these findings.

### 3.5. Virus Isolation and Identification

**Figure 5:**
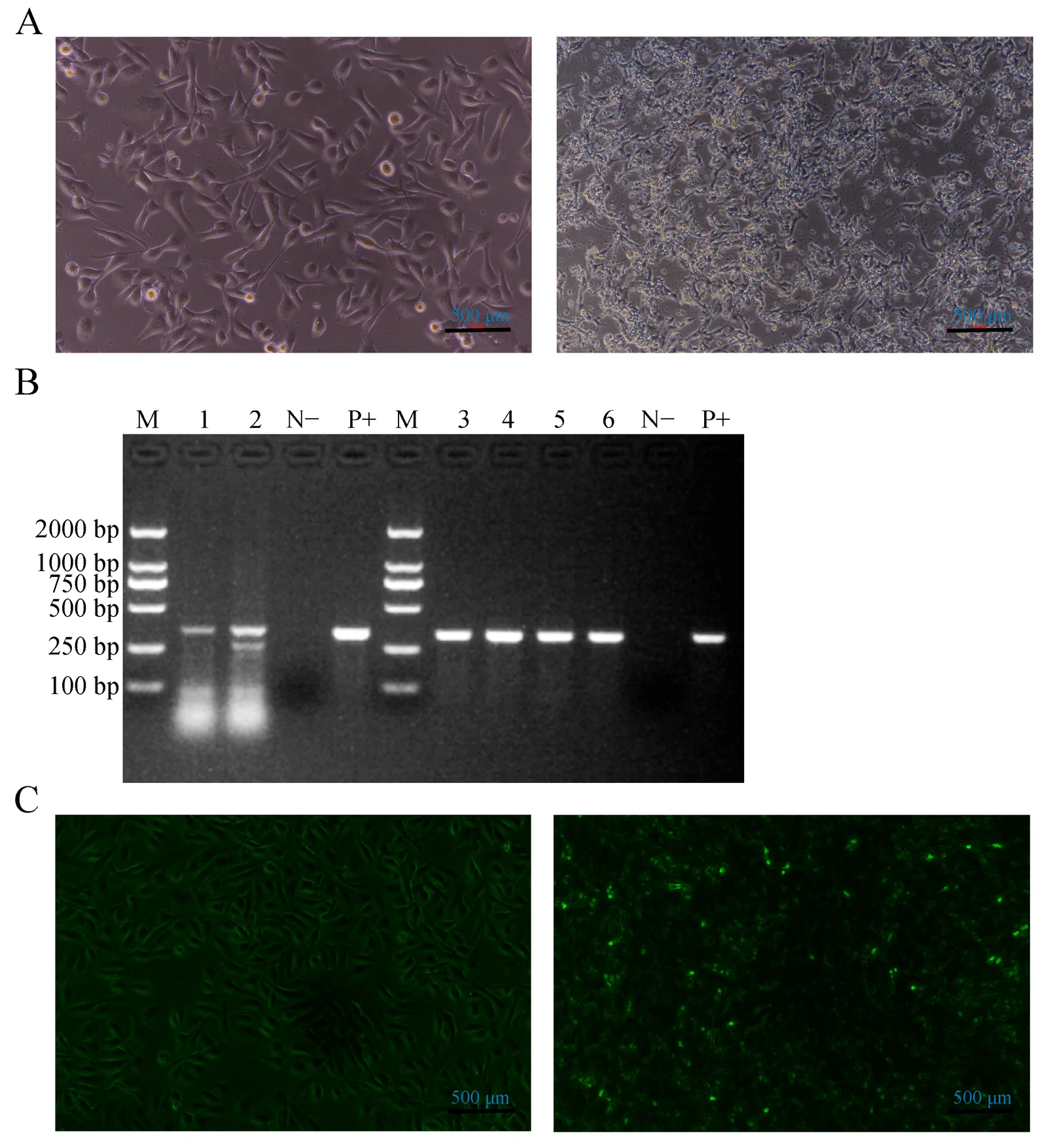
Isolation and identification of the virus. (**A**) Morphological observation of MDBK cells inoculated with the blind-passaged supernatant (scale bar: 500 µm). Left: mock-infected cells (no CPE); right: virus-infected cells (typical CPE). (**B**) PCR detection of viral nucleic acid. Lanes 1 and 2: first-passage samples from isolates #1 and #2, respectively; lanes 3 and 4: second-passage samples from isolates #1 and #2, respectively; lanes 5 and 6: third-passage samples from isolates #1 and #2, respectively. N−, negative control; P+, positive control; M: DNA marker. (**C**) Immunofluorescence assay (IFA) for BVDV antigen. Positive green fluorescence was observed in infected cells, while no signal was detected in the negative control (scale bar: 500 µm).

To obtain viable virus for further characterization, MDBK cells were inoculated with homogenates prepared from BVDV-positive tissue samples. After serial blind passages, obvious cytopathic effect (CPE), characterized by cell rounding, cytoplasmic granulation, nuclear pyknosis, and progressive detachment from the monolayer, became consistently observable at 48–72 h post-inoculation. With successive passages, the CPE appeared progressively earlier and more pronounced, a pattern consistent with viral adaptation and enrichment during in vitro propagation. Following three blind passages, successful virus isolation was confirmed by RT-PCR detection of BVDV RNA in the cell culture supernatants, whereas no amplification was obtained from mock-inoculated control cultures maintained in parallel (Figure 5). The isolated virus, designated as QH-PSYL-2026, was subsequently subjected to whole-genome sequencing to enable comprehensive phylogenetic and molecular.

### 3.6. Viral Genome Sequencing and Phylogenetic Analysis

**Figure 6:**
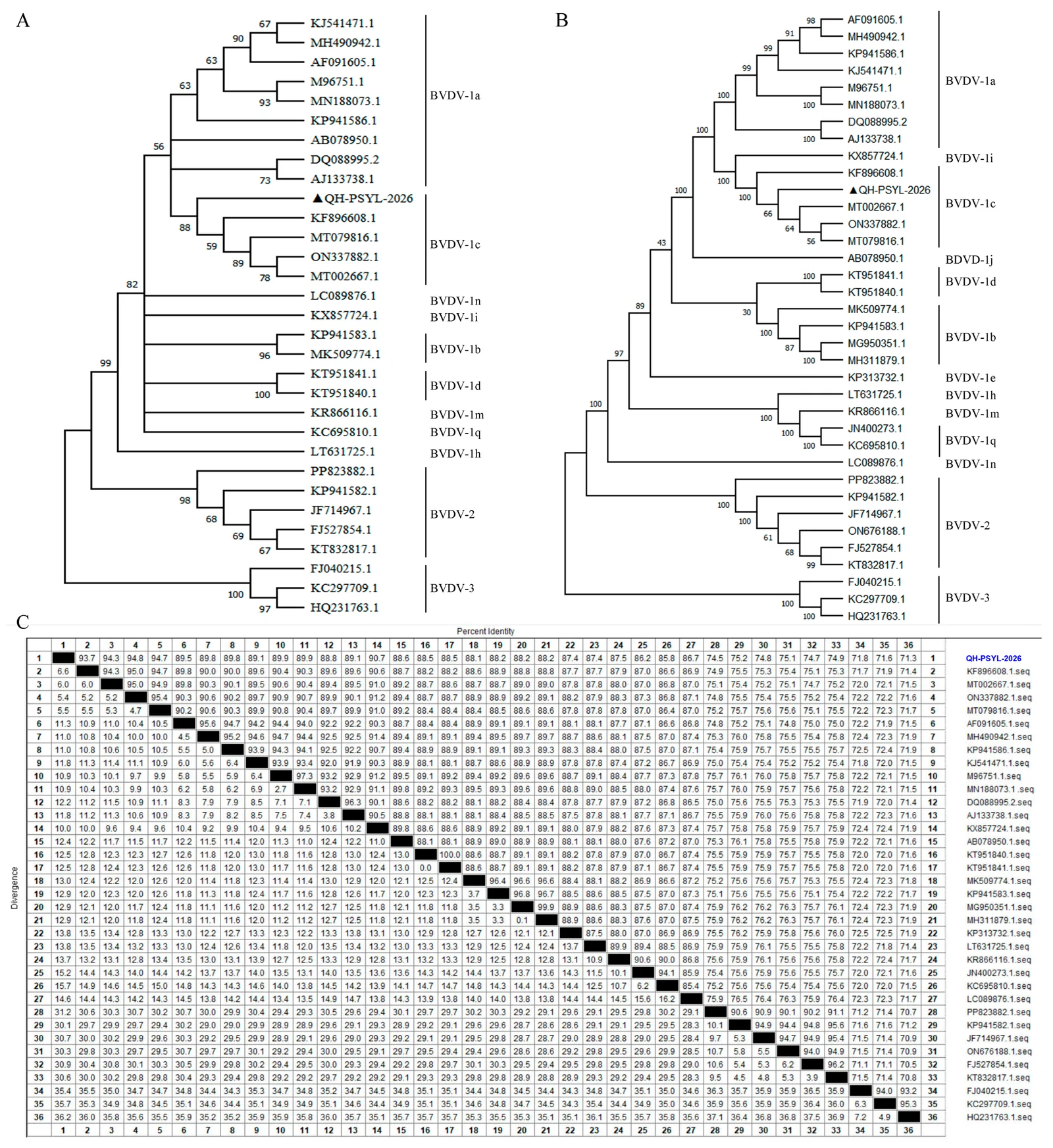
Molecular characterization of the isolated BVDV strain. (**A**) Phylogenetic analysis based on the 5′UTR sequence placed the isolate within the BVDV-1 group, closely related to strains previously reported from livestock in the Qinghai–Tibet Plateau region. (**B**) Whole-genome phylogenetic tree unequivocally assigned the isolate to the BVDV-1c subgenotype. (**C**) Amino acid identity comparisons showed >90% similarity with BVDV-1c reference strains (KF896608.1, MT002667.1, ON337882.1, and MT079816.1) and <90% with other subgenotypes.

Following successful virus isolation, the 5′UTR regions of the viruses recovered from individuals #1 and #2 were amplified and sequenced. Sequence comparison revealed that the two isolates shared an identical 5′UTR sequence (Figure S1). In addition, the 5′UTR sequences amplified directly from the original tissue samples were identical to those of the corresponding cell-culture isolates, further supporting the consistency of the viral sequences between the original samples and the isolated viruses. Therefore, one of the isolates was randomly selected for complete genome sequencing to facilitate further molecular characterization and subtyping. Phylogenetic analysis based on the 5′UTR sequence initially placed the selected isolate QH-PSYL-2026 within the BVDV-1 group and indicated its closest genetic affinity with reference strains previously reported in domestic livestock from the Qinghai–Tibet Plateau region (Figure 6A). To precisely resolve the subgenotype, a robust phylogenetic tree was reconstructed using the complete genome sequence of QH-PSYL-2026, which assigned the isolate to the BVDV-1c subgenotype (Figure 6B, Table S1). Notably, the 5′UTR sequence obtained directly from the original tissue samples was identical to the corresponding region of the complete genome sequence of QH-PSYL-2026, further supporting the consistency of the sequence obtained from the original samples and the isolated virus. The 5′UTR sequence of QH-PSYL-2026 was 100% identical to the 5′UTR sequences of BVDV strains detected from yak samples in our laboratory (Figure S1), and comparative genomic analysis further revealed high amino acid similarity (>90%) between QH-PSYL-2026 and the BVDV-1c reference strains (GenBank accession numbers: KF896608.0, MT002667, ON337882.1, and MT079816.1) (Figure 6C, Table S2). Collectively, these findings provide strong molecular evidence for an interspecies transmission link between domestic livestock and *Procapra przewalskii*. Taken together, the nucleotide-level evidence, corroborated by phylogenetic reconstruction using both the 5′UTR and the complete genome, robustly confirms that QH-PSYL-2026 belongs to the BVDV-1c subgenotype—a subgenotype that has not been previously documented in the Qinghai region [25]. To our knowledge, this is the first molecular evidence of BVDV-1c in Qinghai, offering critical insights into the genetic diversity and local epidemiology of bovine viral diarrhea virus in this high-altitude ecosystem.

Given the cytopathic phenotype observed during virus isolation, the NS2-3 region of QH-PSYL-2026 was further analyzed to investigate potential genetic features associated with cytopathogenicity. Comparative nucleotide sequence analysis of the NS2-3 region revealed no obvious insertions, deletions, duplications, or other large-scale genomic rearrangements in QH-PSYL-2026. Comparison of the deduced amino acid sequence with representative cytopathogenic (cp) and noncytopathogenic (noncp) BVDV strains identified amino acid substitutions at multiple positions in QH-PSYL-2026, including V1154I, L1192S, F1194L, I1200V, V1211I, A1212I, T1228I, L1244I, F1247I, S1256N, M1275R, V1278I, L1280V, V1290I, D1340N, D1364N, S1382G, V1386L, L1394V, L1414M, V1418I, L1429M, D1431E, A1463G, F1471Y, R1487K, S1488N, Y1513H, M1518T, F1545S, I1553V, V1594I, I1612V, I1820V, and L2199I (Figure S2). Among these substitutions, the majority were located within the NS2 region, whereas I1820V and L2199I were located within NS3. Several substitutions were clustered in the C-terminal portion of NS2 and in the region surrounding the NS2-3 junction (Figure S2). Notably, F1545S represented a nonconservative amino acid substitution in the C-terminal region of NS2, where sequence variation was also observed among the analyzed BVDV strains. Because the C-terminal region of NS2 has been implicated in the regulation of NS2-3 processing and the determination of cytopathogenicity in BVDV, the observed sequence differences, particularly those located in this region and around the NS2-3 junction, may represent candidate genetic features potentially associated with the cytopathic phenotype of QH-PSYL-2026. However, these associations remain correlative, and the contribution of individual amino acid substitutions to cytopathogenicity cannot be determined from sequence comparison alone and requires further experimental validation.

## 4. Discussion

*Procapra przewalskii* is a threatened ungulate whose distribution is restricted solely to the area surrounding Qinghai Lake in China [30]. Although conservation efforts have contributed to a gradual increase in population size in recent decades, the species remains threatened by habitat fragmentation, population isolation, reduced genetic diversity, and increasing overlap with domestic livestock grazing areas [2,31]. Previous studies have documented the occurrence of several infectious agents, including parasitic and bacterial pathogens, in wild *Procapra przewalskii* populations, suggesting that infectious diseases may represent an additional challenge to the long-term conservation of this endangered species [32,33]. Furthermore, increased contact between wildlife and livestock can facilitate pathogen spillover at the wildlife–livestock interface, potentially affecting population growth and survival [34].

In this study, we report the first molecular evidence of natural BVDV infection in Przewalski’s gazelle, with the isolated strain QH-PSYL-2026 assigned to the BVDV-1c subgenotype. This finding fills a critical gap in the known host range of BVDV and represents the first documented case of BVDV-associated mortality in this endangered species. As a member of the family Bovidae, Przewalski’s gazelle is phylogenetically closely related to the primary domestic hosts of BVDV, including cattle and sheep, and is therefore considered intrinsically susceptible to infection [35]. This susceptibility is corroborated by previous reports of BVDV infection in diverse wild ruminants, such as white-tailed deer, bighorn sheep, and elk [11,36], suggesting that BVDV possesses a remarkably broad host tropism that frequently extends beyond domestic ungulates.

The Qinghai Lake region, where the *Procapra przewalskii* conservation area is located, is characterized by intensive livestock production, with domestic yak and Tibetan sheep populations exhibiting a notably high seroprevalence of BVDV antibodies [37,38,39]. Persistently infected (PI) livestock, which shed large quantities of virus throughout their lifetime, are widely recognized as the primary sources of BVDV transmission [6,7]. The high genetic identity between QH-PSYL-2026 and local livestock strains—as revealed by both 5′UTR and genomic analyses—provides compelling molecular evidence for a potential interspecies transmission link, and the sympatric grazing patterns and shared grassland resources between free-ranging gazelles and domestic herds likely create multiple opportunities for such spillover events. This high degree of genetic relatedness provides compelling molecular evidence for a potential interspecies transmission link between domestic livestock and Przewalski’s gazelle, and raises the possibility that PI livestock may serve as the source of introduction into the gazelle population.

To our knowledge, this is also the first molecular evidence of the BVDV-1c subgenotype in Qinghai Province. BVDV-1c has been increasingly recognized as a prevalent subgenotype in cattle populations across Asia, including China, Japan, and South Korea, yet its distribution and epidemiological significance in the Qinghai–Tibet Plateau have remained largely unexplored [40,41,42]. The detection of BVDV-1c in an endangered wildlife species in this region not only expands the known geographic range of this subgenotype but also underscores the potential for live-stock-associated viral strains to infect vulnerable wildlife populations. The identical 5′UTR sequences obtained from the original tissue samples and the corresponding cell-culture isolates, as well as their consistency with the corresponding region of the complete genome, further support the reliability of the viral sequence obtained from QH-PSYL-2026. Given that BVDV-1 is strongly associated with several important diseases of cattle, such as bovine respiratory disease, diarrhea and hemorrhagic lesions [43], the prominent pulmonary congestion and gastric hemorrhagic lesions observed in the two gazelles are compatible with lesions that may occur during BVDV infection. Furthermore, the cytopathic effect observed during virus isolation prompted further analysis of the NS2-3 region. Although no obvious insertion, deletion, or duplication was identified, 35 amino acid substitutions were observed compared with representative cp and noncp BVDV strains, including substitutions distributed across the NS2 and NS3 regions (Figure S2). Notably, F1545S was located in the C-terminal region of NS2, and sequence variation was also observed around the NS2-3 junction. These changes may provide molecular clues to the cytopathic phenotype of QH-PSYL-2026, although their specific contribution to cytopathogenicity remains to be determined. Mechanistically, BVDV-induced immunosuppression may predispose affected animals to secondary infections, potentially amplifying tissue damage and accelerating disease progression, although histopathological confirmation is warranted in future cases.

Once introduced into the gazelle population, BVDV could spread within the herd and exert devastating consequences, manifesting as acute mortality, reproductive failure, and immunosuppression that predisposes animals to secondary infections [44]. Considering the small population size, fragmented distribution, and limited genetic diversity of Przewalski’s gazelle, even a single disease outbreak could have catastrophic effects on the long-term viability of this species. Therefore, we strongly recommend that BVDV be incorporated into routine disease surveil-lance programs in conservation areas, with regular screening of both the gazelle population and the surrounding livestock communities, complemented by enhanced biosecurity measures to minimize contact at the wildlife–livestock interface.

Several limitations of this study should be acknowledged. First, the sample size was restricted to two deceased individuals, which precludes a comprehensive assessment of the prevalence, clinical spectrum, and population-level impact of BVDV infection in Przewalski’s gazelle. Second, the lack of serological surveys in the broader gazelle population limits our understanding of the extent of exposure and the potential circulation of BVDV in this species. Third, the absence of longitudinal surveillance data for the local livestock populations precludes a definitive conclusion regarding the directionality of transmission, although the high genetic identity strongly suggests an epidemiological linkage. Fourth, we lacked experimental infection tests, full pathogen screening (bovine parainfluenza virus type 3, bovine coronavirus, bovine respiratory syncytial virus, piroplasms), histopathology, viral immunohistochemistry and tissue viral load tests. Thus we cannot unequivocally attribute the observed pathological lesions solely to BVDV infection, although the consistent detection of viral nucleic acids and the absence of other screened pathogens provide strong supporting evidence. Future investigations with expanded sampling, longitudinal seromonitoring, and comprehensive phylogenetic analyses of both wildlife and livestock strains will be essential to further elucidate the transmission dynamics and epidemiological significance of BVDV in this endangered species.

In conclusion, this study provides the first evidence of natural BVDV-1c infection, virus isolation, and genomic characterization in Przewalski’s gazelle. These findings expand the known host range of BVDV and highlight the emerging threat that livestock-associated viral pathogens pose to the health and conservation of this critically endangered species on the Qinghai–Tibet Plateau. Our results underscore the urgent need for integrated pathogen surveillance and evidence-based management strategies to mitigate the risks of cross-species transmission in this unique alpine ecosystem.

## 5. Conclusions

In conclusion, this study reports the first detection, isolation, and whole-genome characterization of BVDV in *Procapra przewalskii*, identifying the strain as subgenotype BVDV-1c. The complete genome and 5′UTR sequences exhibited extremely high identity with local cattle and yak isolates, providing molecular evidence suggestive of an epidemiological linkage at the wildlife–livestock interface. These findings extend the known host range of BVDV and highlight the potential threat that livestock-associated pathogens pose to this endangered species on the Qinghai–Tibet Plateau. Our results underscore the urgent need to incorporate BVDV surveillance into routine health-monitoring programs for both gazelle populations and surrounding domestic livestock, and to implement enhanced biosecurity measures to reduce potential pathogen transmission risks. This work provides a useful scientific basis for the conservation management of *Procapra przewalskii* and for disease prevention strategies in plateau ecosystems.

## Figures and Tables

**Table 1:** Table of primer information used for detection.

Detection of Target Pathogens	Gene Specificity	Sequence	Product Size (bp)	Annealing Temperature	Ref
*Clostridium perfringens*	Alpha (α) toxin- cpa	GCTAATGTTACTGCCGTTGA	325	53 °C	[17]
		CCTCTGATACATCGTGTAAG			
	Beta (β) toxin- cpb	ACTATACAGACAGATCATTCAACC	236	57 °C	[18]
		TTAGGAGCAGTTAGAACTACAG			
	Epsilon (ε) toxin- etx	ACTGCAACTACTACTCATACTGTG	541	55 °C	[19]
		CTGGTGCCTTAATAGAAAGACTCC			
*Mannheimia haemolytica*	LKT	GCAGGAGGTGATTATTAAAGTGG	207	58 °C	[20]
		CAGCAGTTATTGTCATACCTGAAC			
*Mycoplasma ovipneumoniae*	16SrRNA	TGAACGGAATATGTTAGCTT	361	55 °C	[21]
		GACTTCATCCTGCACTCTGT			
*Klebsiella pneumoniae*	khe	TGATTGCATTCGCCACTGG	428	55 °C	[22]
		GGTCAACCCAACGATCCTG			
*Mycoplasma capricolum* subsp. *capripneumoniae*	arcD	ATCATTTTTAATCCCTTCAAG TACTATGAGTAATTATAATATATGCAA	316	47 °C	[23]
*Pasteurella multocida*	KMT1	ATCCGCTATTTACCCAGTGG	460	55 °C	[24]
		GCTGTAAACGAACTCGCCAC			
BVDV	5′UTR	CTAGCCATGCCCTTAGTAGGACTA	310	55 °C	[25]
		CAACTCCATGTGCCATGTACAGCA			
IBRV	gB	CACGGACCTGGTGGACAAGAAG	656	60 °C	[26]
		CTTCGATCACGCAGTCGCTCA			

## Data Availability

The data presented in this study are available on request from the corresponding authors. The data are not publicly available due to ethical and wildlife conservation considerations related to the protection of Przewalski’s gazelles, as well as restrictions on the use, handling, and management of samples and viral isolates derived from this protected wildlife species.

## Supplementary Materials

The following supporting information can be downloaded at: https://www.mdpi.com/article/10.3390/ani16172781/s1, Figure S1: Sequence alignment of the 5′UTR region of BVDV from *Procapra przewalskii* (#1 and #2) and yaks (Yak-5′UTR), showing 100% identity; Figure S2. Amino acid sequence alignment of the NS2-3 region of QH-PSYL-2026 and representative cytopathogenic (cp) and noncytopathogenic (noncp) BVDV strains. Table S1. Genomic sequencing and basic genomic features of BVDV strain QH-PSYL-2026; Table S2. Detailed information of closely related BVDV-1c reference strains.

## Abbreviations

The following abbreviations are used in this manuscript:

BVDV	Bovine viral diarrhea virus
IBRV	Infectious bovine rhinotracheitis virus
PPRV	Peste des petits ruminants virus
MDBK	Madin–Darby Bovine Kidney
PI	Persistent infection
5′UTR	5′ Untranslated Region
IFA	Indirect Immunofluorescence Assay
CPE	Cytopathic effect
