# Supplementary material for: First Isolation and Genomic Characterization of BVDV-1c in Przewalski’s Gazelle (Procapra przewalskii) from the Qinghai–Tibet Plateau, China
Source: Animals (Basel). 2026 Sep 4;16(17):2781. doi: 10.3390/ani16172781 (PMC13565316; doi:10.3390/ani16172781)
Supplement: Supplementary file 1 [file animals-16-02781-s001.zip › animals-4483993-supplementary.pdf]

# Supporting information

## First Isolation and Genomic Characterization of BVDV-1c in Przewalski's Gazelle (*Procapra przewalskii*) from the Qinghai–Tibet Plateau, China

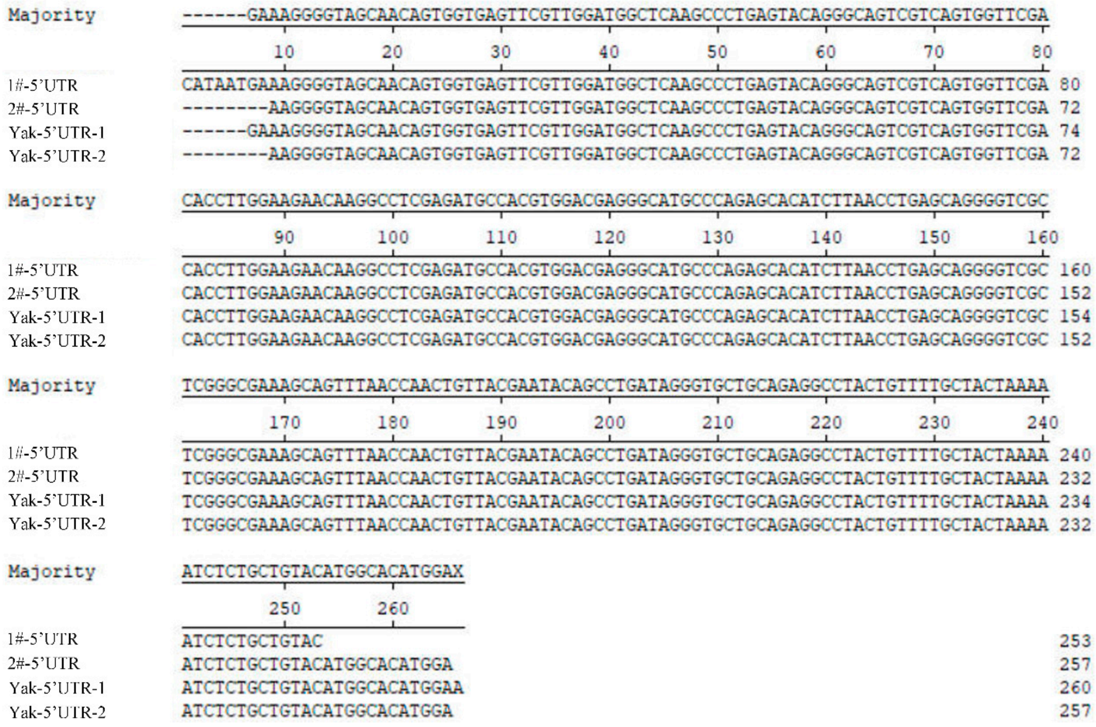


**Figure S1.** Sequence alignment of the 5′ UTR region of BVDV from *Procapra przewalskii* (#1 and #2) and yaks (Yak-5'UTR), showing 100% identity.

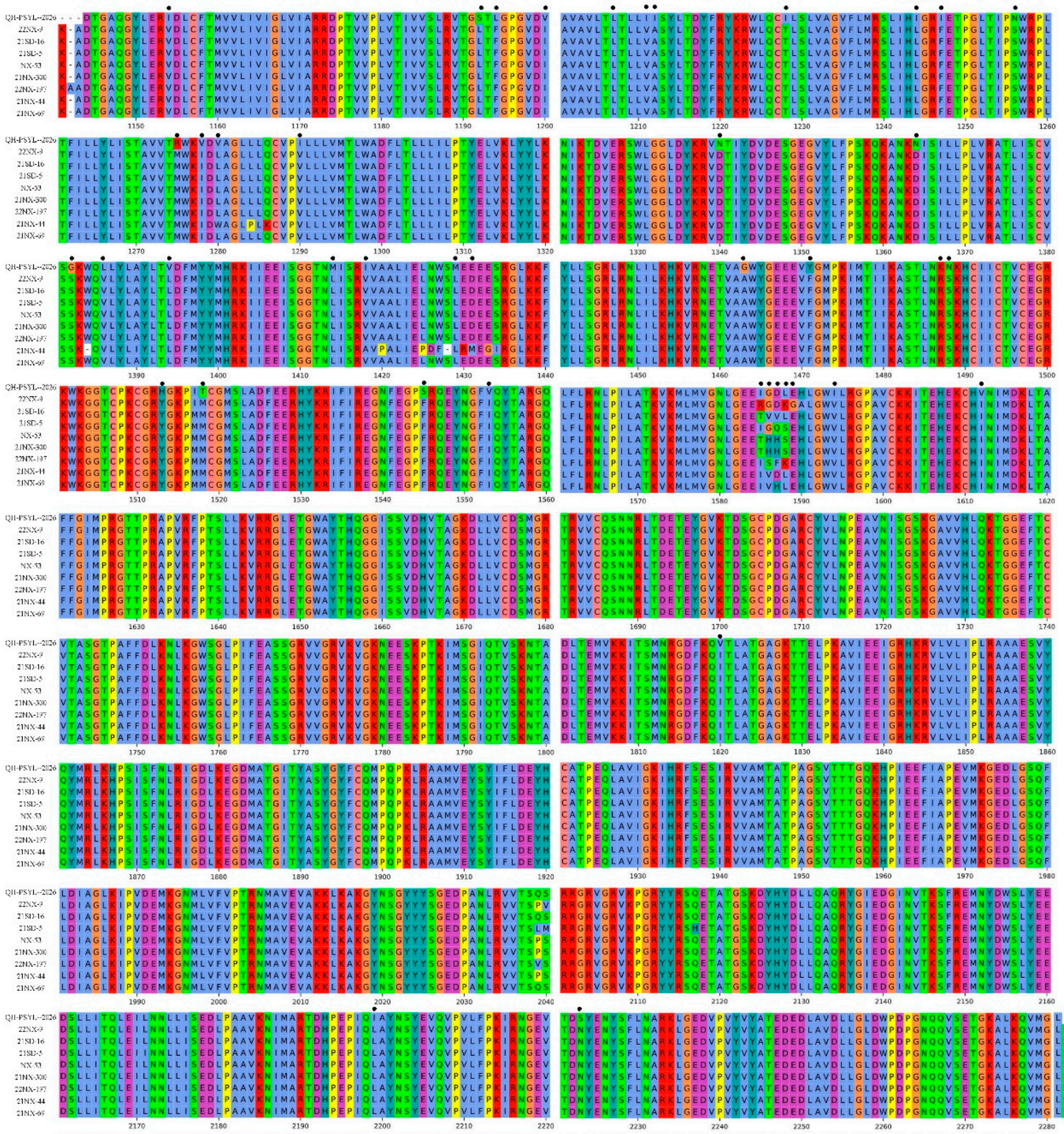

**Figure S2.** Amino acid sequence alignment of the NS2-3 region of QH-PSYL-2026 and representative cytopathogenic (cp) and noncytopathogenic (noncp) BVDV strains.

Amino acid sequence alignment of the NS2-3 region of QH-PSYL-2026 with four representative cp BVDV strains (21NX-53, 21NX-300, 21SD-16, and 21NX-9) and four representative noncp BVDV strains (21NX-69, 21NM-44, 21SD-5, and 22NX-197). Black dots above the alignment indicate amino acid differences among the compared sequences.

**Table S1.** Genomic sequencing and basic genomic features of BVDV strain QH-PSYL-2026

| Index | Value |
|---|---|
| Strain name | QH-PSYL-2026 |
| Host species | Procapra przewalskii |
| Full genome length | 11,819 bp |
| GC content | 45.34% |
| N ratio | 0.01% |
| Total clean reads | 9,668,092 |
| Mapped reads | 962 |
| Genome coverage | 100% |
| Average sequencing depth | 20.51× |

**Table S2.** Detailed information of closely related BVDV-1c reference strains

| GenBank Accession No. | Strain Name | Host | Geographical Origin | Collection Time | Subtype | Genome Length |
|---|---|---|---|---|---|---|
| KF896608.1 | Bega-like | Cattle | Australia | December 2012 | BVDV-1c | 12,193 bp |
| MT002667.1 | 8093 | Cattle | USA | - | BVDV-1c | 12,254 bp |
| ON337882.1 | NM2103 | Cattle | China | March 2021 | BVDV-1c | 12,275 bp |
| MT079816.1 | GXNN1 | Cattle | China | September 2018 | BVDV-1c | 12,277 bp |
